# Dapagliflozin prevents ERK activation and SGLT2‐dependent endoglin upregulation in a mechanically provoked cardiac injury model

**DOI:** 10.14814/phy2.15990

**Published:** 2024-04-04

**Authors:** Tung‐Chen Yeh, Yi‐Chung Wu, Tzyy Yue Wong, Gwo‐Ching Sun, Ching‐Jiunn Tseng, Pei‐Wen Cheng

**Affiliations:** ^1^ Division of Cardiology, Department of Internal Medicine Kaohsiung Veterans General Hospital Kaohsiung Taiwan; ^2^ Institute of Biomedical Sciences National Sun Yat‐sen University Kaohsiung Taiwan; ^3^ Section of Neurology Zuoying Armed Forces General Hospital Kaohsiung Taiwan; ^4^ School of Medicine National Defense Medical Center Taipei Taiwan; ^5^ Department of Medical Education and Research Kaohsiung Veterans General Hospital Kaohsiung Taiwan; ^6^ International Center for Wound Repair and Regeneration National Cheng Kung University Tainan Taiwan; ^7^ Department of Anesthesiology Kaohsiung Veterans General Hospital Kaohsiung Taiwan

**Keywords:** cardiac overloading, cardiomyocytes, cyclic stretch, SGLT2

## Abstract

Sodium‐glucose cotransporter 2 inhibitors (SGLT2i) are rapidly gaining ground in the treatment of heart failure (HF) with reduced ejection fraction (HFrEF) and acute myocardial infarction (AMI) by an unknown mechanism. Upregulation of Na^+^/H^+^ exchanger 1 (NHE1), SGLT1, and Ca^2+^/calmodulin‐dependent protein kinase II (CaMKII) in the diseased hearts was found to be attenuated by prolonged SGLT2i treatment. Unfortunately, dapagliflozin is not well understood as to how Na^+^/Ca^2+^ homeostasis is affected in cardiomyocytes. In this study, we aimed to investigate whether mechanical stretch in cardiomyocytes upregulate SGLT2, resulted to loss of Na^+^/Ca^2+^ homeostasis via ERK and eNOS signaling. AMI (+) and AMI (−) serum levels were estimated using ELISA assays of TGFβ‐1 or endoglin (CD105). Human cardiomyocyte cell line AC16 was subjected to different stresses: 5% mild and 25% aggressive, at 1 Hz for 24 h. Immunofluorescence assays were used to estimate troponin I, CD105, SGLT1/2, eNOS^S633^, and ERK1/2^T202/Y204^ levels was performed for 5% (mild), and 25% elongation for 24 h. AMI (+) serum showed increased TGFβ1 and CD105 compared to AMI (−) patients. In consistent, troponin I, CD105, SGLT1/2, eNOS^S633^ and ERK1/2^T202/Y204^ were upregulated after 25% of 24 h cyclic stretch. Dapagliflozin addition caused SGLT2 inhibition, which significantly decreased troponin I, CD105, SGLT1/2, eNOS^S633^, and ERK1/2^T202/Y204^ under 25% cyclic stretching. In summary, SGLT2 may have sensed mechanical stretch in a way similar to cardiac overloading as in vivo. By blocking SGLT2 in stretched cardiomyocytes, the AMI biomarkers (CD105, troponin I and P‐ERK) were decreased, potentially to rescue eNOS production to maintain normal cellular function. This discovery of CD105 and SGLT2 increase in mechanically stretched cardiomyocytes suggests that SGLT2 may conceive a novel role in direct or indirect sensing of mechanical stretch, prompting the possibility of an in vitro cardiac overloaded cell model, an alternative to animal heart model.

## 1 INTRODUCTION

Coronary artery disease (CAD) has emerged as the leading cause of death worldwide which accounts for approximately 16.7 million deaths per year (Mozaffarian et al., 2015). Acute myocardial infarction (AMI) is key to CAD, when AMI insults the heart, blood flow in one or more of the coronary arteries is blocked, gradually leading to ischemia in the cardiac muscles (Nigam, 2007). The outcome of AMI insult or heart attack may cause heart failure (HF). It occurs with the sudden interruption of coronary blood flow and is a life‐threatening medical emergency which requires quick and immediate treatment (Wang et al., 2019).

A growing number of studies suggest that sodium‐glucose cotransporter 2 (SGLT2) inhibitors exert direct cardioprotective effects (Chen et al., 2022). Recently, SGLT2 inhibitors are implemented in the HF guidelines for European Society of Cardiology (ESC) in heart failure patients having preserved ejection fraction (HFpEF) (McDonagh et al., 2021). This recommendation is independent for any existing diabetes mellitus despite the history of this drug being classified as anti‐diabetic. This reflects the ongoing reconceptualization of the molecular mechanisms for SGLT2 inhibitors beyond their glucosuric effects (Packer, 2019). Various cardiac mechanisms, such as ion homeostasis, redox status, inflammation, and metabolism, have been found to be directly regulated by SGLT2i within cardiomyocytes. These mechanisms are deregulated during HF, which results in endothelial dysfunction, arrhythmias, sudden cardiac death, diastolic dysfunction, fibrosis, and reduced cardiac performance, most of which may be alleviated by SGLT2i therapy. According to previous evidence (Chung et al., 2021; Pabel et al., 2020), this effect is possibly caused by direct SGLT2i regulation of cardiac plasma membrane ion exchangers, channels, and cooperators. These ion exchangers, channels, and cotransporters transport sodium into cells. The effect of SGLT2i on heart is evident primarily in diseased hearts, but not in healthy hearts. The reason is because sodium transporters are more active in pathological conditions (hyperglycemia, mechanical overload, hypertension, obesity, systemic inflammation, ischemia) compared to healthy hearts. SGLT2i has proven to be effective in reducing morbidity and mortality in patients with HF and hypertension. Nevertheless, more research is needed to understand the effects of SGLT2i on the heart of patients with healthy and diseased hearts. Interestingly, treatment with dapagliflozin (6 weeks) attenuated Ca^2+^ and Na^+^ overload and increased Ca^2+^ transient amplitude in HFpEF animal model that consumed high salt diet, which is also known as Dahl salt‐sensitive rats (Cappetta et al., 2020). Upregulation of Na^+^/H^+^ exchanger 1 (NHE1), SGLT1, and Ca^2+^/calmodulin‐dependent protein kinase II (CaMKII) in the diseased hearts was found to be attenuated after prolonged dapagliflozin treatment. Unfortunately, it is not well understood as to how dapagliflozin affects Na^+^/Ca^2+^ homeostasis in cardiomyocytes.

In vivo study has shown that cardiac overloading is a form of excessive mechanical stimulation which led to oxidative stress, increased ERK protein expression (Fiorillo et al., 2005), and increased nitric oxide (NO) (Tirziu & Simons, 2008) as indicators for hypertrophy. ERK inhibitor abolished the effect of empagliflozin, indicating that ERK may be a potential downstream target (Hu et al., 2021). The endothelial nitric oxide synthetase (eNOS) is expressed in the cardiovascular (CV) cells and cardiomyocytes (Cotton et al., 2002), and eNOS is dysfunctional during cardiac hypertrophy (Flaherty et al., 2007). When eNOS is dysfunction during cardiac hypertrophy, β‐adrenergic receptor (β‐AR) showed compensatory function to the abnormal eNOS. Furthermore, overexpression of eNOS is linked to decreased cardiac contractile function which affected survival rate in congestive HF patients (Jones et al., 2003). Surprisingly, cardiac cells with decreased contractility survived through the cardiac overloading via an unknown mechanism. Besides, clinical studies have revealed that the expression of endoglin, a kind of accessory protein for the TGF‐β1 receptor, is increased in patients having atherosclerotic disease (Ikemoto et al., 2012). Cardiac troponins and CK‐MB are equally sensitive biomarkers during the first 48 h of AMI. AMI diagnosis can therefore be established based on these biomarkers as early as 1.5–3 h after the onset of symptoms prior to AMI (de Winter et al., 1995). Based on these previous findings, we aimed to determine whether: (1) TGF‐β1 and CD105 may be the new indicators for detecting mechanically overloaded cardiomyocytes; (2) cyclic stretch may upregulate SGLT 2, which results to loss of Na^+^/Ca^2+^ homeostasis via ERK and eNOS signaling upregulation. Discerning the underlying mechanism for cardiomyocyte upon sensing mechanical cues in its microenvironment is essential for developing pathologically relevant cardiac disease models. In this study, we investigated the impact of pathologically relevant 25% elongation on SGLT2 function in AC16 human cardiomyocyte cell line, compared to mild cyclic stretch of 5% elongation. Our findings path a way to understanding of SGLT2 function in CVD.

## 2 MATERIALS AND METHODS

### 2.1 Ethics statement

This study follows the guidelines of the Declaration of Helsinki with approval from the Institutional Review Board (IRB number VGHKS19‐CT3‐16) at Kaohsiung Veterans General Hospital, Kaohsiung, Taiwan. All collected specimens were anonymized prior to analysis. The subjects identified as having AMI were based on ICD codes. AMI proclamation was based on the indexed date when AMI was first clinically diagnosed, such that the date for AMI onset was identical to the date of AMI proclamation. Exclusion criteria included acute or chronic infections or inflammatory diseases, severe hepatic or renal dysfunction, malignant tumors, or hematologic disorders. The CAD was divided into two groups, namely patients without AMI (Group 1) and patients with AMI (Group 2). The source of samples was derived from the biobank.

### 2.2 Cell culture

Human cardiomyocytes AC16 were purchased from MERCK (Catalogue #SCC109, Temecula, CA, USA). Cells were cultured in DMEM/F12 (Simply, CC115‐0500) in culture medium supplemented with penicillin and streptomycin (Thermo Fisher Scientific, 15240‐062) and 10% fetal bovine serum (Millipore, TMS‐013‐BKR). The cells were incubated in 5% CO_2_, humidified atmosphere at 37°C. Cells were passaged every 3–4 days; passages 2–10 were used in this study.

### 2.3 Cyclic stretch

The stretch device is ATMS Boxer, purchased from TAIHOYA Corporation (Taiwan, R.O.C.). The ATMS Boxer is placed in a CO_2_ incubator maintained at 37°C. The stretch device is composed of chambers that accommodate cells on the polydimethylsiloxane (PDMS) membrane (TAIHOYA Corporation, Taiwan, R.O.C.). The PDMS surface was coated with quick coating solution collagen I (ANGIO‐PROTEOMIE, cAP‐01, source: porcine) before seeding cells at density 4 × 10^4^ per cm^2^. Based on a previous study on moderate and cardiac overloading model (Wong et al., 2018), the cells were subjected to cyclic stretching of 5%, 25% elongation at 1 Hz for 24 h.

### 2.4 ERK and SGLT2 inhibitors

ERK inhibitor PD98059 (Merck KGaA, Darmstadt, Germany, P215) or SGLT2 inhibitor DAPA (Combi‐Blocks, San Diego, CA, QE‐4375) was purchased and prepared in ultrapure double distilled water. Cells were treated with PD98059 (30 μM) or DAPA (25 μM) while still under cyclic stretching for 24 h.

### 2.5 Immunofluorescence assay

Cells were fixed in 4% paraformaldehyde for 20 min, washed in PBS, then incubated in Triton X‐100 for 10 min (0.5% v:v). Next, cells were blocked in 5% (w:v) bovine serum albumin for 30 min, washed using PBS, then incubated with primary antibody overnight at 4°C. Troponin I antibody (GeneTex; GTX113028), P‐ERK antibody (Cell Signaling; CS4370S), P‐eNOS^S633^ (BD 612665), SGLT1 (Abcam; ab14686) and SGLT2 (Abcam; ab85626) were used in this study. After hybridization with primary antibodies, cells were incubated in secondary antibody, Alexa Fluor‐conjugated anti‐rabbit and anti‐mouse (1:200, Invitrogen; A11029, A11031, A11034, A11036) for 1 h. Finally, cells were stained and mounted in the Prolong® Diamond Antifade Mounting Medium which contains DAPI (Abcam, ab104139). All images acquired with Olympus BX51 microscope equipped with an Olympus DP74 camera and an UplanFL N 20X objective (Olympus) using the CellSens acquisition software.

### 2.6 Human endoglin/CD105 assay

To analyze CD105 and TGF‐β level, the human CD105 ELISA kit (Boster Biological Technology, CatEK0644) and human TGF‐β assay ELISA kit (Boster Biological Technology, CatEK0513) were used to detect CD105 and TGF‐β level, respectively, in the collected serum. The assay was performed immediately after supernatant of serum was collected, or immediately after cell pellet was collected. ELISA was performed in 96‐well plates, and the absorbance was measured at 575 nm.

### 2.7 Statistical analysis

Data were analyzed with Mann–Whitney *U* test. Correlations were assessed using Spearman's rank correlation moment correlation coefficient. All results are shown as median (interquartile range). All measurements were generated independently at least three times. Significant effects were subsequently investigated using the *p*‐values for normally distributed data. Differences are considered statistically significant when *p* < 0.05.

## 3 RESULTS

### 3.1 Characteristics of patients

The main demographic and clinical characteristics of all participants are summarized in Table 1. CAD patients were divided into two groups by pathological types: AMI (−) and AMI (+). There was no difference between AMI (−) and AMI (+) groups in risk factors such as sex, age, DM, and hypertension (Table 1).

**TABLE 1:** Demographic details for participants in this study.

Variables	CAD patients		
	AMI (+) (*n* = 22)	AMI (−) (*n* = 27)	*p*‐value
	No. (%)	No. (%)	
Sex			
Female	3 (13.6)	5 (18.5)	0.715^a^
Male	19 (83.4)	22 (81.5)	
Age, years (mean ± SD)	62.9 ± 10.7	61.2 ± 10.8	0.580^b^
≦65	13 (59.1)	17 (63.0)	0.782^c^
>65	9 (40.9)	10 (37.0)	
DM			
No	11 (50.0)	18 (66.7)	0.238^c^
Yes	11 (50.0)	9 (33.3)	
HTN			
No	10 (45.5)	11 (40.7)	0.740^c^
Yes	12 (54.5)	16 (59.3)	

^a^ *p*‐value estimated by Fisher's exact test.

^b^ *p*‐value estimated by Student's *t*‐test.

^c^ *p*‐value estimated by the chi‐squared test.

### 3.2 AMI biomarkers TGF‐β1 and CD105 were activated

**FIGURE 1:**
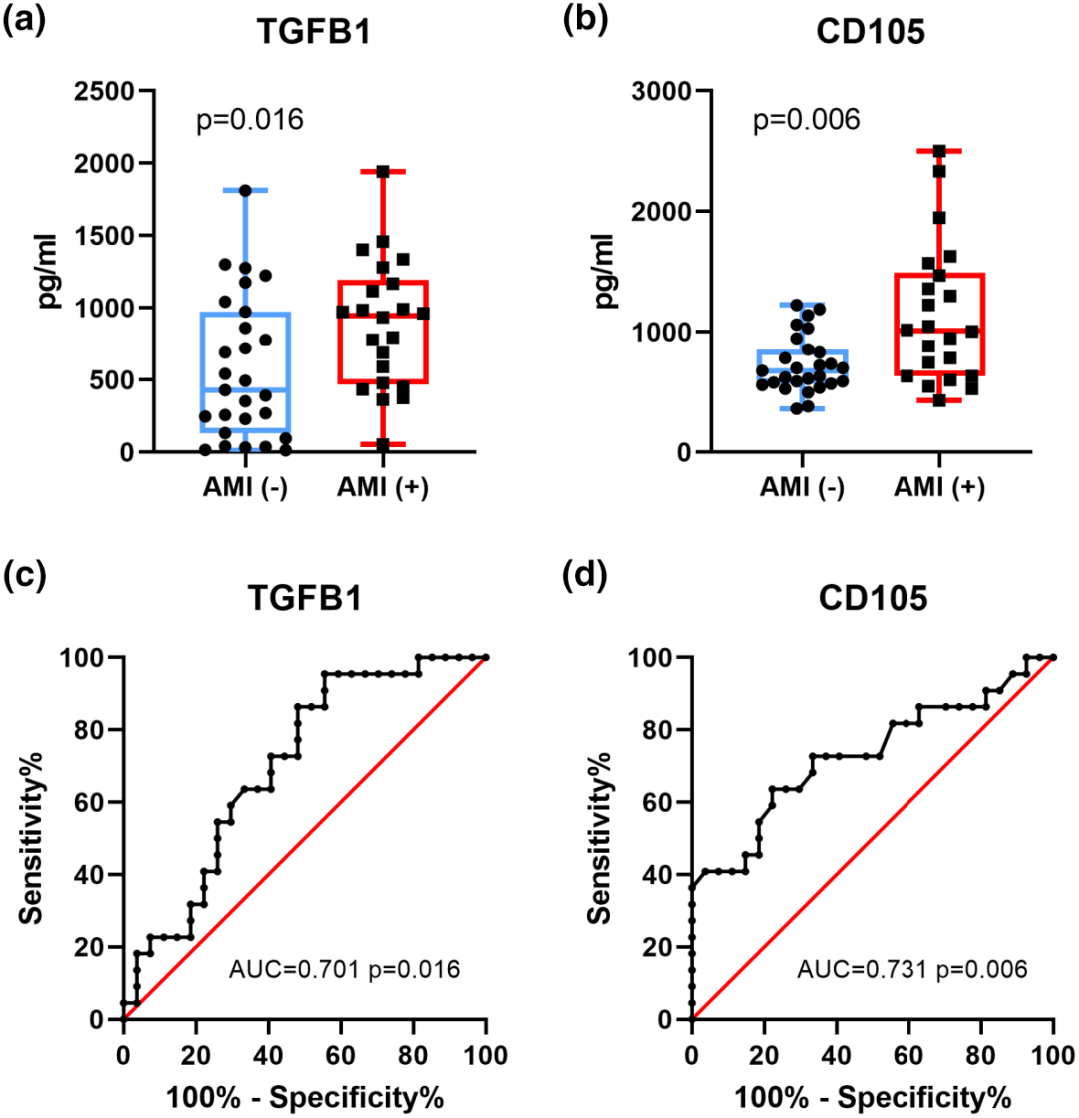
TGF‐β1 and CD105 demonstrated as CAD biomarkers. (a, b) Bar graphs representing serum levels of TGF‐β1 and CD105 with different CAD severity. (c, d) Receiver operating characteristic (ROC) curve and area under the curve (AUC) for TGF‐β1 and CD105 (AUC = 0.701 and 0.731 respectively). Diagonal line indicates zero predictive value for the model. Data were analyzed with the Mann–Whitney *U* test. Correlations were assessed using Spearman's rank correlation moment correlation coefficient.

AMI (+) patients (*n* = 22) showed increased serum TGF‐β1 and CD105 levels compared to AMI (−) group (*n* = 27) (Figure 1a,b). To assess the discriminatory ability of the models, the model obtained from the derivation sample was applied to the test and validation samples, the area under the Receiver operating characteristic curves (AUCROC) was determined for all datasets. The AUC indicated that both TGF‐β1 (AUCROC: 0.701, *p* = 0.01) and CD105 (AUCROC: 0.731, *p* = 0.006) were the more potent biomarkers for AMI (Figure 1c,d). Several biochemical markers for detecting early myocardial damage have been proposed which includes troponin, CK (creatine kinase) and CK‐MB (CK‐mass). Among other biochemical indices, cardiac troponins are as sensitive as CK‐MB during the first 48 h after onset of AMI. Based on these indicators, AMI diagnosis can be established as early as 1.5–3 h after the onset of symptoms (de Winter et al., 1995). To explain a potential mechanism for TGF‐β1 and CD105, we evaluated their relationship with the AMI‐related biomarkers, such as CK, CK‐MB, and troponin I. Results showed that CD105 significantly correlated with CK (*p* = 0.014), CK‐MB (*p* = 0.003) and troponin I (*p* = 0.032), respectively, whereas troponin I significantly correlated with CK (*p* < 0.001), and CK‐MB (*p* < 0.001). However, TGF‐β1 showed no significant correlation with CK, CK‐MB and troponin I (Table 2). This result suggests that both cytokines TGF‐β1 and CD105 were linked to AMI pathogenesis.

**TABLE 2:** Spearman's rank correlation coefficient for serum TGF‐β1 and CD105, and the different parameters assigned in this study.

Variables	TGFB1 (pg/mL)		CD105 (pg/mL)	
	*r*	*p*‐value	*r*	*p*‐value
CK (U/L)	−0.272	0.221	0.515	0.014
CK‐MB (U/L)	−0.316	0.152	0.601	0.003
Troponin I (ng/mL)	−0.412	0.064	0.469	0.032

### 3.3 Stretching induced mechanical overloading in cardiomyocytes that led to increased AMI risks

**FIGURE 2:**
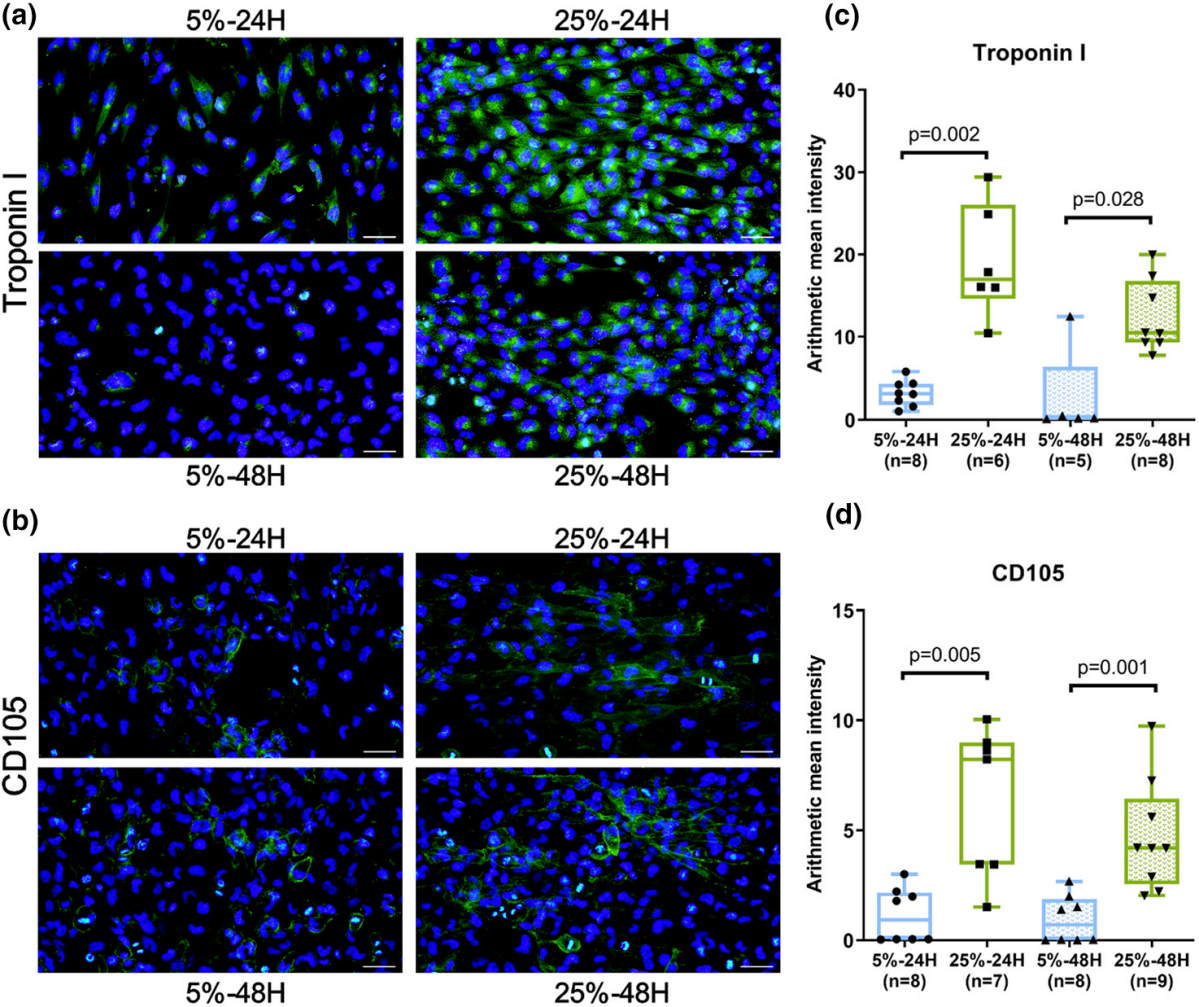
Troponin I and CD105 increased in cardiomyocytes after mechanical stretch. (a, b) Representative images showing troponin I and CD105‐positive cells in human cardiomyocytes after 24 and 48 h cyclic stretch. Cells were counterstained in 4,6‐diamidino‐2‐phenylindole. Scale bar = 50 μm. (c, d) Bar graphs representing troponin I and CD105 fluorescence intensity. Data represented at least three independent experiments in median (interquartile range). Data were analyzed with the Mann–Whitney *U* test.

To test the hypothesis that mechanical stretch indeed promoted mechanical overloading in cardiomyocytes, cells were subjected to stretching strains of 5% and 25%. The 5% strain mimicked the frequency of normal physiological heartbeat, whereas 25% mimicked mechanical overloading. The cardiomyocyte characteristic marker troponin I expression was higher at 25% compared to 5% after 24 h (*n* = 6, *p* = 0.02, Mann–Whitney *U* test) and 48 cyclic stretch (*n* = 8, *p* = 0.028, Mann–Whitney *U* test) (Figure 2). CD105 protein expression was also higher at 25% compared to 5% after 24 h (*n* = 7, *p* = 0.005, Mann–Whitney *U* test) and 48 h cyclic stretch (*n* = 9, *p* = 0.001, Mann–Whitney *U* test) (Figure 2). Cardiac biomarkers are released in the circulation due to damage or death of cardiac myocytes and measuring these biomarkers in serum or plasma is therefore useful for MI diagnosis. In addition, troponin I is a well‐established biomarker for myocardial necrosis (Forough et al., 2011). troponin I and CD105 are characteristic biomarkers in muscle cells and were expressed in AC16. Increased expression of troponin I and CD105 implied that the two cytokines may be the new indicators for detecting mechanically overloaded cardiomyocytes in vitro. As a result, we established a mechanical overloading injury model using cardiomyocytes.

### 3.4 Stretching upregulated SGLT2 protein expression, promoted p‐ERK translocation into nucleus, and increased eNOS expression in cardiomyocytes

**FIGURE 3:**
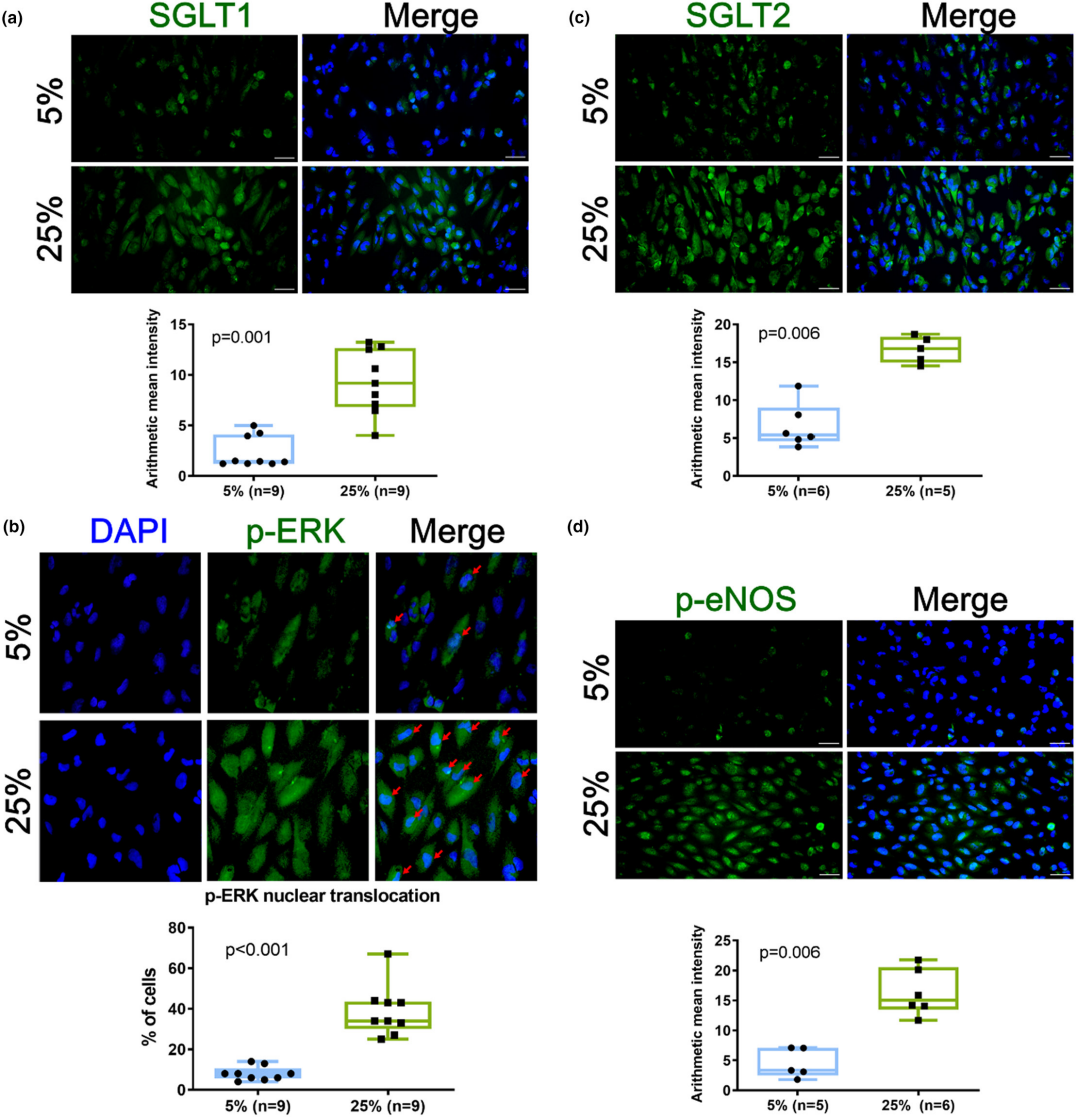
Mechanical stretch upregulated SGLT1/2 protein levels, promoted nuclear translocation of p‐ERK, and induced p‐eNOS expression. (a–d) Green fluorescence indicating SGLT1, SGLT2, p‐ERK, and p‐eNOS‐positive cells in cardiomyocytes after 24 h of 5%, 25% cyclic stretch. Cells were counterstained in 4,6‐diamidino‐2‐phenylindole to exhibit blue fluorescence. Scale bar = 50 μm. Bar graphs representing SGLT1, SGLT2, p‐ERK, and p‐eNOS fluorescence intensity. Data represented at least three independent experiments in median (interquartile range). Data were analyzed with the Mann–Whitney *U* test.

Aside from being an intracellular mediator of extracellular signals, ERK activation is critical for cardiac hypertrophy and HF progression (Wang, 2007). To propose a potential mechanism for SGLT2 and ERK, we evaluated their relationship with mechanical overloading after cyclic stretch. Immunofluorescence staining showed that SGLT1 (*n* = 9, *p* = 0.01), SGLT2 (*n* = 5, *p* = 0.06), and p‐eNOS (*n* = 6, *p* = 0.06) were significantly increased at 25% compared to 5% 24 h cyclic stretch (Figure 3). Besides, immunofluorescence staining demonstrated that nuclear p‐ERK and its mediated transcriptional activity significantly increased and activated under 25%, 24 h cyclic stretch (*n* = 6, *p* = 0.06) (Figure 3b).

### 3.5 SGLT2 and ERK inhibitor restored troponin I and CD105 expressions in cardiomyocytes

**FIGURE 4:**
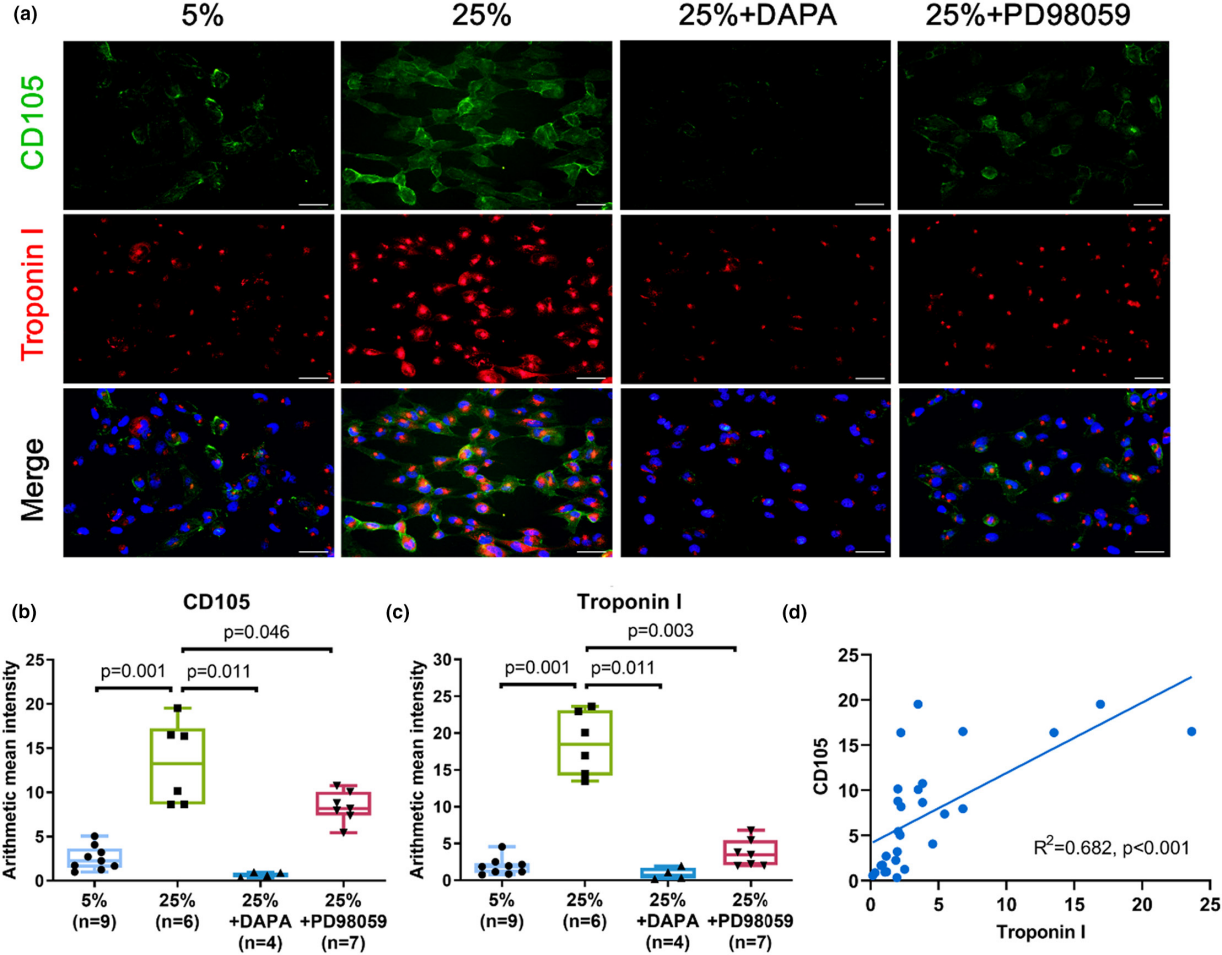
SGLT2 inhibitor decreased troponin I and CD105 protein expressions. (a) Representative images showing green fluorescence‐labeled troponin I and CD105‐positive cells in cardiomyocytes. Cardiomyocytes were treated with DAPA (SGLT2 inhibitor) or PD98059 (ERK inhibitor), followed by 25%, 24 h cyclic stretch. Scale bar = 50 μm. (b, c) Bar graphs showing troponin I and CD105 fluorescence intensity in the cardiomyocytes. (d) Correlation of troponin I and CD105 with cyclic‐stretch‐induced mechanical overloading was assessed. Data represented at least three independent experiments in median (interquartile range). Data were analyzed with the Mann–Whitney *U* test. Correlations were assessed using Spearman's rank correlation moment correlation coefficient.

**FIGURE 5:**
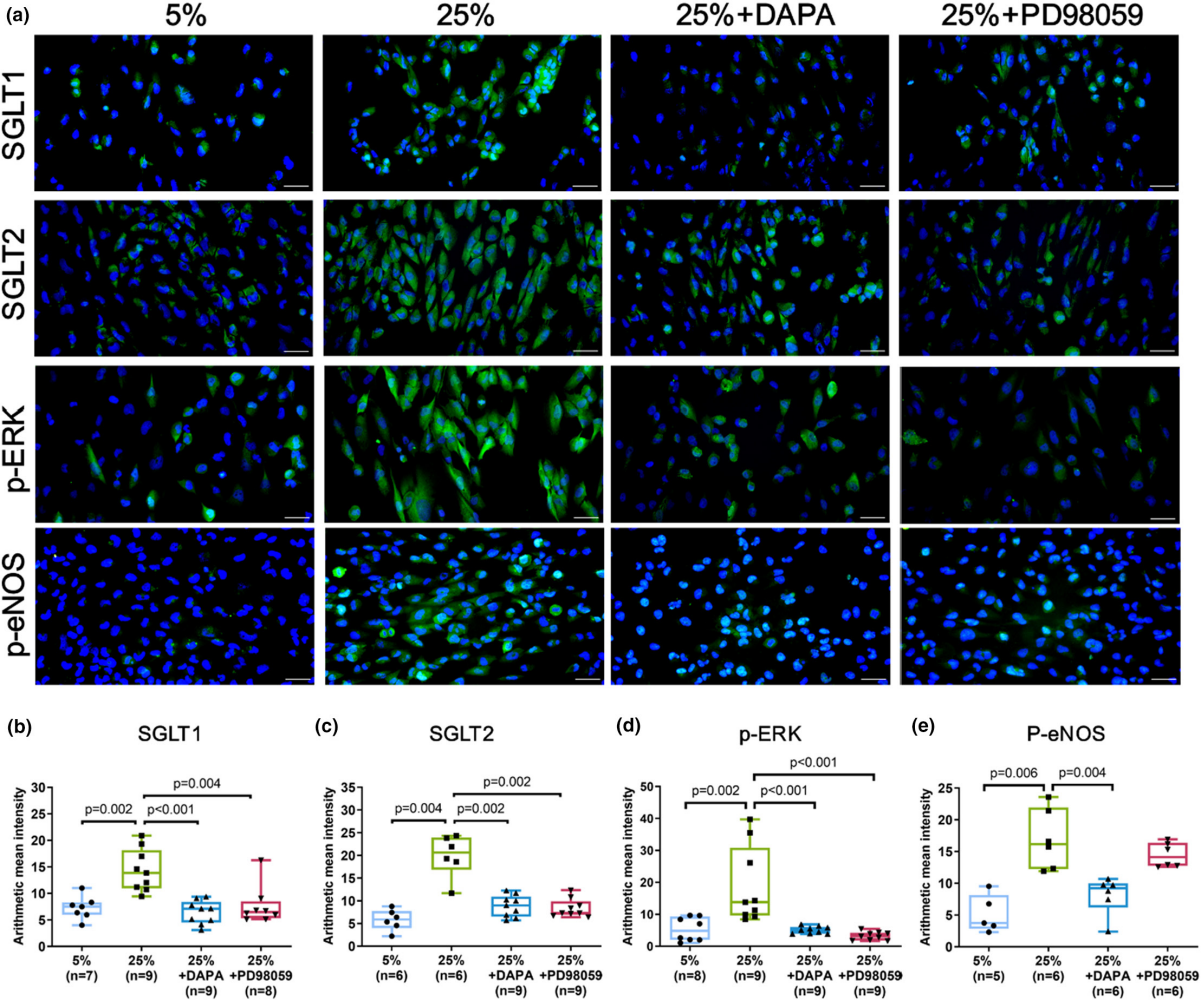
Mechanical stretch induced SGLT1/2 expression, upregulated ERK and eNOS activation. (a) Fluorescence images showing cardiomyocytes positive for SGLT1/2, p‐ERK, and p‐eNOS. DAPA (SGLT2 inhibitor) or PD98059 (ERK inhibitor) were added to cells, and stretched at 5%, 25% for 24 h. Scale bar = 50 μm. (b–d) Quantifications of SGLT1/2 and p‐ERK. (e) p‐eNOS fluorescence intensity in the stretched cells. Data represented at least three independent experiments in median (interquartile range). Data were analyzed with the Mann–Whitney *U* test.

Furthermore, the feasibility of the cardiac physiological and pathological models was assessed by treating cells with the cardiac drug, DAPA (SGLT2 inhibitor). The cells were given either 25 μM DAPA or 30 μM PD98059 (ERK inhibitor). During cardiac overloading, the transport of p‐ERK in and out of cells is changed, and we observed that troponin I (*n* = 6, *p* = 0.001, Mann–Whitney *U* test) and CD105 (*n* = 6, *p* = 0.001) were significantly increased in cytoplasm at 25% strain. However, troponin I in the cytoplasm were significantly decreased when SGLT2 (*n* = 4, *p* = 0.011) and ERK was inhibited (*n* = 7, *p* = 0.003) (Figure 4a–c). Besides, CD105 in the cytoplasm were also significantly decreased when SGLT2 (*n* = 4, *p* = 0.011) and ERK was inhibited (*n* = 7, *p* = 0.046) (Figure 4a,b). Data showed a very significant correlation between AMI and concentration of troponin I and CD105 (Table 2). Here, it was demonstrated that troponin I significantly correlated with CD105 (*p* < 0.001) as a result of mechanical stretch which posed potential mechanical injury to cardiomyocytes (Figure 4d). To investigate if the ERK signaling was activated through SGLT, SGLT2 inhibitor (DAPA) was added to the cells. We observed that SGLT1 (*n* = 9, *p* = 0.002) was significantly increased in cytoplasm at 25% strain. However, SGLT1 in the cytoplasm was significantly decreased when DAPA (*n* = 9, *p* = 0.001) and PD98059 (ERK inhibitor) (*n* = 8, *p* = 0.004) was added (Figure 5a,b). SGLT2 (*n* = 6, *p* = 0.004) in cytoplasm was significantly increased at 25% strain, and SGLT2 in cytoplasm was significantly decreased in presence of DAPA (*n* = 9, *p* = 0.002) and PD98059 (*n* = 9, *p* = 0.002) into the cells (Figure 5a–c). Interestingly, we observed that p‐ERK (*n* = 9, *p* = 0.002) in the cytoplasm was significantly increased at 25% strain, and p‐ERK in the cytoplasm was significantly decreased when DAPA (*n* = 9, *p* < 0.001), and PD98059 (*n* = 9, *p* < 0.001) was added (Figure 5a–d). p‐eNOS (*n* = 6, *p* = 0.006) in the cytoplasm were significantly increased at 25% strain; however, p‐eNOS in the cytoplasm was significantly decreased when DAPA was added (*n* = 6, *p* = 0.004) (Figure 5a–e). Previous study showed that eNOS overexpression promoted survival rate of patients with congestive HF but decreased the cardiac contractile function (Jones et al., 2003).

## 4 DISCUSSION

From a clinical perspective, HF occurrences due to cardiac remodeling after AMI are still on the rise regardless of new treatment or technology that deals with coronary artery, ischemia‐related stenosis with primary percutaneous coronary intervention (PCI). Development of specific cardiac biomarkers may enhance the clinician's ability to detect cell death of cardiomyocytes, and improve detection of cardiac overloading. Therefore, the development of highly sensitive and specific cardiac biomarkers such as cardiac troponin I concentration or CK‐MB fraction activity has greatly enhanced the clinician's ability to detect cell death of cardiomyocytes (Berezin & Berezin, 2020). In recent years, circulating extracellular matrix (ECM) biomarkers such as transforming growth factor (TGF)‐ β1 are useful for analyzing molecular activity during cardiac repair following AMI (Frangogiannis, 2017). Clinical studies have revealed that the expression of the accessory protein for TGF‐β1 receptor, also known as endoglin, is increased in patients having atherosclerotic disease (Ikemoto et al., 2012). Endoglin is expressed in tissues such as mouse and human atherosclerotic vascular endothelial cells and smooth muscle cells (Jain et al., 2005). Expression of endoglin is related to eNOS in endothelial cells (which can repair the blood vessel wall), plaque neovascularization, collagen production, and atherosclerotic plaque damage (Santibanez et al., 2007). Cardiac troponins and CK‐MB are equally sensitive biomarkers during the first 48 h of AMI onset. AMI diagnosis can therefore be established based on these biomarkers as early as 1.5–3 h after the onset of symptoms (de Winter et al., 1995). Our current study indicates that both TGF‐β1 and CD105 can be applied as biomarkers for detecting AMI (Figure 1). Therefore, increased troponin I and CD105 implied that the two cytokines may be novel indicators for detecting mechanically overloaded cardiomyocytes. As a result, we had established a mechanical overloading injury in vitro model using cyclic stretch.

Previous studies have identified numerous risk factors for AMI‐related heart attack, including diabetes mellitus, hypertension, hypercholesterolaemia, smoking, alcohol consumption, obesity, and sedentary lifestyle (Oppenheimer, 2010; Wilson et al., 1998). Hypertensive heart can induce HF and is characterized by mechanical pressure overloading greater than 140 mmHg (King, 2007). Mechanical forces such as stretching, bending, and compression are able to regulate cardiac cellular structure and function (Peyronnet et al., 2016). Previous studies demonstrated that mechanically activated proteins may have role in overcoming cardiac overloading; however, this has not been studied in cardiac cells. In vivo study has shown that cardiac overloading is a form of excessive mechanical stimulation, demonstrated that mechanical stretching led to oxidative stress, increased ERK protein expression (Fiorillo et al., 2005) and NO (Tirziu & Simons, 2008) which are indicators for hypertrophy. The endothelial eNOS is expressed in the CV cells and cardiomyocytes (Cotton et al., 2002), and eNOS is dysfunctional eNOS during cardiac hypertrophy (Flaherty et al., 2007). In addition, β‐AR showed compensatory function to abnormal eNOS during cardiac hypertrophy. Furthermore, overexpression of eNOS is linked to decreased cardiac contractile function which affected survival rate in congestive HF patients (Jones et al., 2003). Surprisingly, cardiac cells with decreased contractile function survived through the cardiac overloading through an unknown mechanism.

In cardiomyocytes, Na^+^ homeostasis is finely orchestrated by activity of several transporters, ion channels and kinases. Cardiomyocyte is fundamental for cardiac tissue function and Na^+^ homeostasis is required for excitation‐contraction coupling and normal mitochondrial metabolism. HF is characterized by cellular Na^+^ overload as a consequence of imbalance between Na^+^ influx and efflux. Increase in Na^+^ influx is predominantly caused by: (1) an increase in late Na^+^ current (Valdivia et al., 2005), (2) increase in Na^+^/H^+^ exchanger (NHE1) activity and (3) increase in Na2^+^/Ca2^+^ exchanger (NCX) forward mode due to cytosolic Ca^2+^ overload in cardiomyocytes (Despa & Bers, 2013). Given the role of Na^+^ homeostasis for cardiac excitation‐contraction coupling as well as cardiac metabolism, there is no surprise that dysregulation of Na^+^ is central to development and progression of HF. Sodium‐glucose co‐transporter 2 inhibitors (SGLT2i) are the latest treatment strategy for HF. SGLT2i is known to improve ejection fraction (HFrEF), also known as the DELIVER and EMPEROR‐Preserved. SGLT2i has potential to become the first drug to improve CV disease with preserved ejection fraction (HFpEF) and HFrEF, especially in patients suffering from HF (Trum et al., 2021), but the underlying mechanism is still unclear.

Aside from the possible relationship between SGLT2 and Na^+^ influx in cardiac‐related disease, SGLT2 has been linked to cardiac myocyte activity. Previous studies on SGLT2 in cardiac myocytes have been contrasting and there exists disagreement on the function of SGLT2 in cardiac myocytes. SGLT2 mRNA has been investigated in single cell sequencing of left ventricular myocytes from human hearts (Di Franco et al., 2017), as well as cardiomyocytes derived from human‐induced pluripotent stem cells (hiPSCs) (Ng et al., 2018). Transversal ex vivo studies on SGLT2 expression (Di Franco et al., 2017; von Lewinski et al., 2010) have also been investigated in human cardiomyocytes. However, these studies were not explicitly designed to evaluate the presence of SGLT2 protein in cardiomyocytes. Moreover, Marfella et al., investigated whether SGLT2 is expressed in failing human hearts of diabetic and non‐diabetic patients, and in AC16 human cardiomyocyte cell line. Previously, a prospective study with a follow‐up of patients that had underwent first heart transplant (HTX) had been evaluated. Explanted heart, basal (1 week after HTX), and final (48 weeks after HTX) endomyocardial biopsies (EMBs) from patients were evaluated for SGLT2 presence in cardiomyocytes by immunohistochemistry, immunofluorescence and SGLT2 quantification of real‐time reverse transcription‐polymerase chain reaction and western blot analysis. Our evidence showed that that SGLT2 protein was highly expressed in cells from diabetic and non‐diabetic end‐stage failing hearts. Furthermore, SGLT2 mRNA level was highly expressed in cells from end‐stage failing hearts. Moreover, the FISH analysis showed high level of SGLT2 mRNA in cardiomyocytes, suggesting that SGLT2 protein may possess crucial function in cardiac myocytes from diabetic and nondiabetic end‐stage failing hearts. Interestingly, healthy implanted hearts in diabetic patients showed a progressive increase in cardiomyocyte's SGLT2 expression within 1 year. Thus, the diabetic milieu can promptly increase the cardiomyocyte SGLT2 expression, whereas the same effects were not seen in healthy hearts implanted in non‐diabetic patients (Marfella et al., 2022). Recent research indicates that certain pathological conditions stimulate SGLT2 expression. For example, increase in angiotensin II and oxidative stress stimulates SGLT2 expression in porcine coronary artery endothelial cells (Park et al., 2021). During the first few days after myocardial infarction, SGLT2 may be transiently expressed in ischemic part of the heart (Lee et al., 2021). When this is indeed the case, SGLT2 may also contribute to higher cytoplasmic sodium ions since sodium transporters are mostly activated during pathological conditions such as hyperglycemia, mechanical overloading, hypertension, obesity, systemic inflammation, and ischemia. Troponin I is a well‐established biomarker for myocardial necrosis (Forough et al., 2011), and CD105 is a characteristic marker expressed by muscle cells. In cardiomyocytes, cyclic stretching increased the expression of troponin I and CD105, which may be indicating a breach, an injury in cardiac cells (Figure 2).

We observed that mechanical injury through stretching upregulated SGLT1 and SGLT2 protein expressions, translocation of p‐ERK into nucleus and increased eNOS expression in the cardiomyocytes (Figure 3). Here, we demonstrated that mechanical stretch stimulated increased expression of troponin I and CD105 through activation of SGLT1 and/or SGLT2. Although there is disagreement in regard to the presence or absence of SGLT2 in cardiac myocytes, the presence of SGLT2 in cardiomyocytes and the metabolic effects related to its silencing, offers new insights to understanding the indirect or direct beneficial effects of SGLT2i on cardiac cells.

The anti‐arrhythmic drugs mentioned above have been in clinical use for decades, whereas SGLT2 inhibitors are relatively new player in this field. To examine the potential anti‐arrhythmic effects of SGLT2 inhibitors, we ought to look beyond a direct interaction with the electrical conduction system of the heart. So far, anti‐arrhythmic properties for SGLT2 inhibitors are not known and have not been investigated. Our data showed that troponin I and CD105 in cytoplasm were significantly increased at 25% elongation, and significantly decreased when SGLT2 and ERK were inhibited (Figure 4a–c). Consistently, SGLT1/2, p‐ERK, and p‐eNOS were decreased when SGLT2 was inhibited, and ERK was inhibited at 25% elongation (Figure 5). As for the role of ERK during this dynamic response, ERK was involved in development of cardiac hypertrophy and progression of HF (Wang, 2007). The eNOS overexpression promoted survival rate of patients having congestive HF, but cardiac cells of patients had decreased contractile function (Jones et al., 2003). At present, cardiac cells are speculated to have survived cardiac overloading with decreased contractile function through unknown mechanism.

**FIGURE 6:**
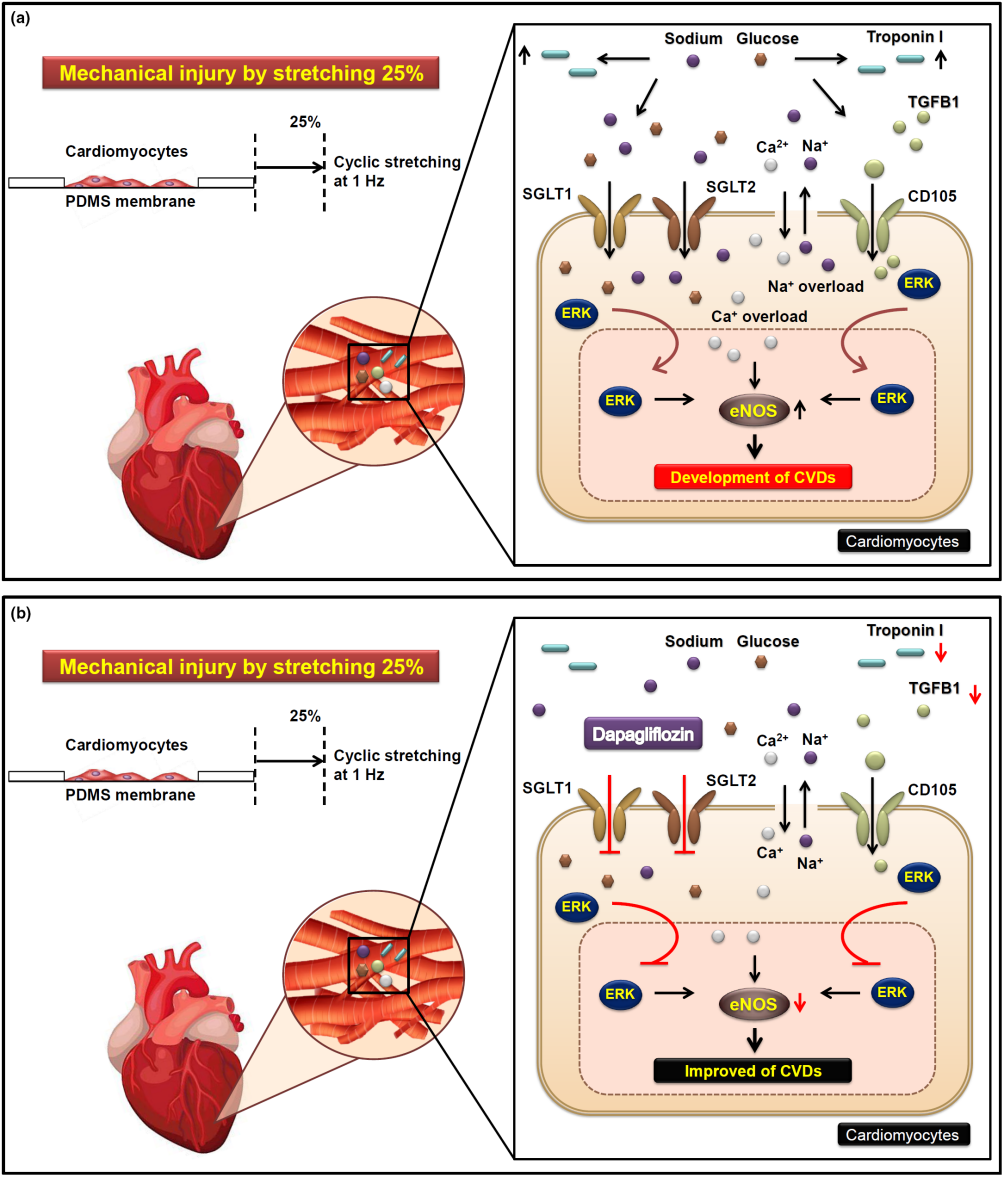
Proposed mechanism for Dapagliflozin on troponin I, endoglin (CD105) and ERK activation in the mechanically injured cardiac cell model. Our model depicts the upregulation of troponin I and endoglin (CD105) after mechanically triggered cardiac injury. (a) Cardiomyocytes were seeded on stretchable PDMS membrane, subjected to cyclic stretch at 5% and 25% elongation. After cyclic stretch, troponin I and CD105 were increased, triggering downstream p‐ERK to enter cell nucleus, potentially altered gene transcription that promoted upregulation of p‐eNOS. The accumulated p‐ERK nuclear translocation and p‐eNOS activation led to an imbalanced regulation of sodium/calcium ions. Finally, the cardiomyocytes were overwhelmed by loss of sodium/calcium ions homeostasis, renting the cells susceptible to CVD‐related risk factor. (b) Dapagliflozin (DAPA), the inhibitor having higher affinity for SGLT2, is able to prevent the characteristics of the mechanically provoked biomarkers for cardiac injury. As a result, SGLT2 and/or SGLT1 is crucial in maintaining the normal function of cardiomyocytes.

Human AC16 cells were developed in 2005 by Davidson et al. (2005). Their quick propagation in culture and homogeneous response to stimuli make them suitable for high‐throughput drug screening experiments, as well as modeling CV diseases, including cardiomyopathies, hypertrophic responses, adverse remodeling, metabolic changes, and ischemia–reperfusion injuries (Hoes et al., 2019). Cardiac cell lines might be useful and valuable tools in CV research. However, there are numerous limitations that may impact the results obtained with these in vitro cell culture experiments. Therefore, care must be taken when choosing the ideal model when considering the characteristics of these cell lines. To eliminate these disadvantages, specific protocols are available to induce differentiation of these cells toward a more mature cardiac phenotype. Nonetheless, there are only few studies characterizing the phenotype of these cell lines in their differentiated stage (Davidson et al., 2005; Fukushima et al., 2018). In contrast, primary cell cultures, in particular human iPSC‐derived cardiomyocytes may be better suited for in vitro studies compared to cell lines due to their higher degree of similarity to adult cardiac tissue. The ones that more closely resemble myocardial gene expression, such as human‐induced pluripotent stem cell‐derived cardiac myocytes (hiPSC‐CMs) are used only in a minority of publications. Indeed, human iPSC‐derived cardiomyocyte cells account for approximately 15%–18% of PubMed records searching by keywords including “human,” “induced pluripotent stem cell” and “heart” out of the analyzed cell lines including all type of cell lines and primary cultures. Although kits for iPSC‐derived cardiomyocyte generation are commercially available, numerous challenges for maintaining iPSC‐derived cardiomyocyte still persist. Furthermore, there are variable origins of initiating cells and diverse differentiation protocols when generating hiPSC‐CMs that complicates the comparative evaluation of different cell cultures. Aside from the high variability among hiPSC‐CMs, they are frequently immature and show distinct characteristic compared to mature cardiomyocytes, which may lead to unsuccessful in vitro*‐to*‐in vivo translation (Denning et al., 2016; Tu et al., 2018). Their limited availability may further complicates then as in vitro platform for high‐throughput drug screening. Further studies should be designed to assure that the in vitro conclusions can be extrapolated to in vivo processes. As a result, the detailed knowledge of strengths as well as limitations of the cardiac cell lines is essential. Mechanical forces such as stretching, bending and compression can regulate cardiac cellular structure and function (Peyronnet et al., 2016). Previous studies demonstrated that mechanically activated proteins may have role in overcoming cardiac overloading, and this has not been studied in cardiac cells. Here, we showed that cardiomyocytes may have maintained their function through SGLT2 in regulation of SGLT1/2, p‐ERK, and p‐eNOS expression levels. The major findings from this study include: (1) TGF‐β1 and CD105 are closely linked to AMI risk factors including CK, CK‐MB, and troponin I; (2) Specific cardiac biomarkers (troponin I and CD105) were expressed in the cardiomyocytes and were increased after 25% cyclic stretch; (3) Cyclic stretch may have upregulated cardiac plasmalemmal ion exchangers/cotransporters (NHE‐1, SGLT), resulted to loss of Na^+^/Ca^2+^ homeostasis via ERK and eNOS signaling upregulation. Prolonged ERK and eNOS signaling upregulation may further promote accumulation of troponin I and TGF‐β1 (CD105) in cardiomyocytes, which ultimately led to abnormal cardiac cells. Furthermore, SGLT1 or SGLT2 inhibition helped reduced troponin I and TGF‐β1 (CD105) in cardiomyocytes, possibly through maintaining Na^+^ and Ca^2+^ homeostasis (Figure 6).

## 5 CONCLUSIONS

Our findings support the idea that SGLT2i is indeed the rising star for treating patients with CAD. A multifaceted approach is required to improve our knowledge on the pathophysiologic basis of ischemic cardiac injury. The molecular signals involved in death of cardiac cells, the role of mechanical stress, the relationship between mechanical stress and ischemia‐related injury, these factors are difficult to be examined on animals because of variations among individual animals, time‐consuming and the high cost of animal experiments. Nevertheless, the molecular events can be routinely tested on in vitro cell model for different types of cardiac cells and with different cardiac‐related drugs. In the future, we would like to examine the mechanism related to dysrhythmias (including HF) following onset of myocardial infarction.

## 6 CLINICAL PERSPECTIVES

1. TGF‐β1 and CD105 are the molecular link to AMI and its risk factors including CK, CK‐MB, and troponin I.
2. SGLT2 antagonists have considerable clinical potential in diabetes‐induced cardiovascular (CV) complications and diabetes because they reduce renal glucose reabsorption and have protective effects on the heart, by an unknown mechanism.
3. Cyclic stretch upregulated glucose, resulted to loss of Na^+^/Ca^2+^ homeostasis via ERK and eNOS signaling upregulation. It is plausible to explain that prolonged ERK and eNOS signaling upregulation may further promote the accumulated troponin I and TGF‐β1 (CD105) in cardiomyocytes, which ultimately leads to cardiac‐related diseases.
4. Inhibition of SGLT1 and/or SGLT2 was able to protect cardiomyocytes from prolonged increased ERK and eNOS activation, possibly preventing cardiomyocytes from developing into diseased cardiomyocytes such as that found in hearts of AMI.

## AUTHOR CONTRIBUTIONS

Conceptualization of this study was contributed by Tung‐Chen Yeh, Gwo‐Ching Sun, and modified by Professor Yi‐Chung Wu, Tzyy Yue Wong. Data organization, writing, and editing were performed by Pei‐Wen Cheng. Supervision and interpretation were contributed by Ching‐Jiunn Tseng and Pei‐Wen Cheng.

## FUNDING INFORMATION

This project was supported by Kaohsiung Veterans General Hospital (VGHKS108‐103, VGHKS109‐082, KSVGH110‐062, KSVGH112‐066, KSVNSU‐113‐009, KSVGH‐113‐056) and Zuoying Armed Forces General Hospital (KAFGH‐ZY_A_111001, KAFGH‐ZY‐A‐112005, KAFGH‐ZY_A_113017) were the project codes.

## CONFLICT OF INTEREST STATEMENT

The authors of this study declared no competing interest.

## ETHICS STATEMENT

The study was conducted strictly following guidelines of the Declaration of Helsinki with approval from the Institutional Review Board at Kaohsiung Veterans General Hospital (Kaohsiung, Taiwan; IRB number: VGHKS19‐CT3‐16).

## INFORMED CONSENT

All collected human data and specimens were anonymized before analysis. As a result, written informed patient consent was waived by the Institutional Review Board at Kaohsiung Veterans General Hospital.

## CONSENT FOR PUBLICATION

Not applicable.

## Data Availability

The data that support the findings of this study are available from the corresponding author upon reasonable request.
